# Extremely preterm infants born outside a provincial tertiary perinatal center and transferred postnatally associated with poor outcomes: a real-world observational study

**DOI:** 10.3389/fped.2024.1287232

**Published:** 2024-02-13

**Authors:** Sicong Peng, Xianjing He, Shiwen Xia

**Affiliations:** ^1^ Department of Neonatology, Maternal and Child Health Hospital of Hubei Province, Tongji Medical College, Huazhong University of Science and Technology, Wuhan, Hubei, China; ^2^ Clinical Research Center of the Neonatal Emergency Medicine of Hubei Province, Wuhan, Hubei, China; ^3^ Neonatal Emergency Transfer Center of Hubei Province, Wuhan, Hubei, China

**Keywords:** extremely preterm infants, in-utero transfer, outcome, birth asphyxia, periventricular-intraventricular hemorrhage, extrauterine growth retardation, treatment

## Abstract

**Introduction:**

Extremely preterm infants (EPIs) have high morbidity and mortality, and are recommended to be born in a tertiary perinatal center (inborn). However, many EPIs in central China are born in lower-level hospitals and transferred postnatally, the outcomes of which remain to be investigated.

**Methods:**

EPIs admitted to the Department of Neonatology, Maternal and Child Health Hospital of Hubei Province from January 2013 to December 2022 were retrospectively recruited and divided into the control (inborn) and transfer groups (born in other hospitals). The neonatal and maternal characteristics, neonatal outcomes, and the treatment of survival EPIs were analyzed.

**Results:**

A total of 174 and 109 EPIs were recruited in the control and transfer groups, respectively. EPIs in the transfer group have a higher birth weight and a lower proportion of multiple pregnancies than the control group (all *P* < 0.05). The proportions of antenatal steroids, magnesium sulfate, cesarean delivery, premature rupture of membranes ≥18 h, gestational diabetes, and amniotic fluid abnormalities were lower in the transfer group (all *P* < 0.05). Survival rates (64.22% vs. 56.32%), proportions of severe periventricular-intraventricular hemorrhage (PIVH) (11.93% vs. 11.49%), severe bronchopulmonary dysplasia (sBPD) (21.05% vs. 20%), and severe retinopathy of prematurity (ROP) (24.77% vs. 20.11%) were similar in the transfer and control groups (all *P* > 0.05). However, the transfer group had higher proportions of severe birth asphyxia (34.86% vs. 13.22%, *P* < 0.001), PIVH (42.20% vs. 29.89%, *P* = 0.034), and extrauterine growth retardation (EUGR) (17.43% vs. 6.32%, *P* = 0.003). Less surfactant utilization was found in the transfer group among survival EPIs (70.00% vs. 93.88%, *P* < 0.001).

**Conclusion:**

EPIs born outside a tertiary perinatal center and transferred postnatally did not have significantly higher mortality and rates of severe complications (severe PIVH, severe ROP, and sBPD), but there may be an increased risk of severe asphyxia, PIVH and EUGR. This may be due to differences in maternal and neonatal characteristics and management. Further follow-up is needed to compare neurodevelopmental outcomes, and it is recommended to transfer the EPIs *in utero* to reduce the risk of poor physical and neurological development.

## 1 Introduction

Extremely preterm infants (EPIs, gestational age <28 weeks) have high mortality and morbidity due to the extreme immaturity of multiple organs, such as the lungs, brain, digestive system, etc. As a result, these infants are susceptible to complications such as periventricular-intraventricular hemorrhage (PIVH), bronchopulmonary dysplasia (BPD), retinopathy of prematurity (ROP), necrotizing enterocolitis (NEC), and extrauterine growth retardation (EUGR) (1, 2). A recent multicenter study reported that the number of EPI admissions to Neonatal Intensive Care Units (NICUs) in China increased from 2010 to 2019, with an incidence of severe complications up to 72.4% and regional differences (3). The clinical outcomes of EPIs depend on whole-chain management from pregnancy, delivery room, and NICU to the family, and often need multidisciplinary medical resources (4). Moreover, improper early postnatal resuscitation and treatment, such as inadequate preparation, irregular procedures, or inappropriate equipment, may have a great impact on the survival rate and long-term prognosis of EPIs (5, 6).

Medical resources in mainland China are not well distributed, and the availability of therapeutic modalities varies considerably among NICUs of different sizes (7). Thus, the recent Chinese expert consensus suggested that pregnant women at risk of delivering EPIs undergo an in-utero transfer to a provincial tertiary perinatal center (4). However, many EPIs are born and treated in lower-level hospitals where postnatal transfers are performed, and their outcomes and management remain unclear. Although previous studies have reported that EPIs born in non-tertiary hospitals had higher mortality and rates of severe brain injury, NEC, focal intestinal perforation, and cognitive impairment (8–10), there is a lack of comparison of outcomes and treatment between inborn and transfer EPIs in China. We hypothesized that the clinical outcomes and treatment of EPIs differed between inborn and transfer EPIs. Thus, this study aims to conduct the above-mentioned comparison through a real-world retrospective study, in order to provide support to the practice of in-utero transfer of EPIs.

## 2 Methods

### 2.1 Patients

EPIs admitted to the Department of Neonatology, Maternal and Child Health Hospital of Hubei Province from January 2013 to December 2022 were retrospectively recruited. Exclusion criteria: (1) repeated records of the same patients (two consecutive medical records of the same patients were integrated and counted as one case); (2) incomplete records (the patients were still in the hospital at the time of manuscript submission); (3) records of EPIs readmitted to the hospital for other diseases (pneumonia, jaundice, anemia, etc.) after discharge. The EPIs in the control group were born in the Maternal and Child Health Hospital of Hubei Province, a provincial tertiary perinatal center in China. Meanwhile, the EPIs in the transfer group were born in lower-level hospitals and transferred postnatally.

### 2.2 Protocol for the transfer of EPIs

All EPIs were transferred according to the routine of our NICU as follows. Before transfer, the local hospital was instructed on how to perform the necessary examination and treatment. During the transfer, an ambulance-mounted incubator was used and STABLE technology was applied (11, 12). Briefly, blood Sugar and body Temperature were maintained in an appropriate range, Airway was kept unobstructed and oxygen was provided as needed, Blood pressure was monitored, the necessary Laboratory tests were performed, and Emotional support was provided to family members (11, 12). The Neonatal Pain/Agitation and Sedation Scale (N-PASS) was used to evaluate pain in infants undergoing mechanical ventilation or other painful procedures, and fentanyl was administered when the N-PASS score was >3 (13, 14). Additionally, for EPIs within 7 days of life, in order to minimize the incidence of PIVH, the head and limbs of the infants were immobilized with the restraint belt equipped with the incubator to maintain the midline position during transfer.

### 2.3 Data collection and definition

The neonatal and maternal characteristics, neonatal outcomes, and the treatment of survival EPIs were analyzed. Birth asphyxia was diagnosed as reported (15). Mild asphyxia was diagnosed when a 1-min Apgar score or 5-min Apgar score was ≤7, with umbilical artery blood gas pH <7.2; and severe asphyxia was diagnosed when a 1-min Apgar score ≤3 or 5-min Apgar score was ≤5, with umbilical artery blood gas pH <7.0. Apgar score was used to diagnose neonatal asphyxia when umbilical artery blood gas analysis was unavailable, and other causes of low Apgar score, such as congenital malformations of the respiratory, circulatory, or central nervous system, neuromuscular disorders, fetal hemorrhagic shock, fetal edema, and fetal passive drug poisoning caused by high doses of anesthetic analgesics during labor, were excluded in clinical practice to reduce misdiagnosis (15). The definition and severity of BPD were based on the NICHD criteria published in 2001 (16). In addition, infants who had died of respiratory failure between 14 days of life and 36 weeks postmenstrual age (PMA) were classified as having severe BPD (sBPD) (17). The diagnosis of PIVH is based on brain B-ultrasound and MRI findings (18). Complete treatment refers to those infants who received active treatment through discharge (including survival at discharge and in-hospital death), excluding those EPIs whose treatment was withdrawn by parental decision. During the hospitalization of all completely treated EPIs, brain B-ultrasound was routinely monitored at least once within the first 7 days of life, then re-examined if the results were abnormal. At least one brain MRI was routinely performed before the 36th week of PMA or at discharge. Moderate to severe hypothermia was defined as an infant admission temperature ≤35.9 °C (19). EUGR was defined as a weight below the 10th percentile of the Fenton growth chart at 36 weeks PMA or at discharge (2, 20). Discharge criteria for EPIs (21): (1) PMA ≥ 34 weeks; (2) vital signs are stable, no apnea; (3) completion of oral/intestinal feeding and well tolerated; body weight ≥2,000*g* with stable growth; (4) maintaining normal body temperature at room temperature; (5) recovery of illness, or family sequential treatment is feasible and safe; (6) caregivers are able and ready to care.

### 2.4 Statistical analysis

IBM SPSS Statistics software (version 26.0) was used for statistical analysis. The categorical variables were presented as numbers and proportions (%), and the chi-squared test with or without continuous correction was used to compare the proportions of the two groups, if applicable. Numerical variables were expressed as mean ± standard deviation or median (interquartile range). The unpaired Student's *t* test was used to compare the numerical variables between the two groups. *P* values less than 0.05 were considered statistically significant.

## 3 Results

### 3.1 Characteristics of the EPIs

**Figure 1:**
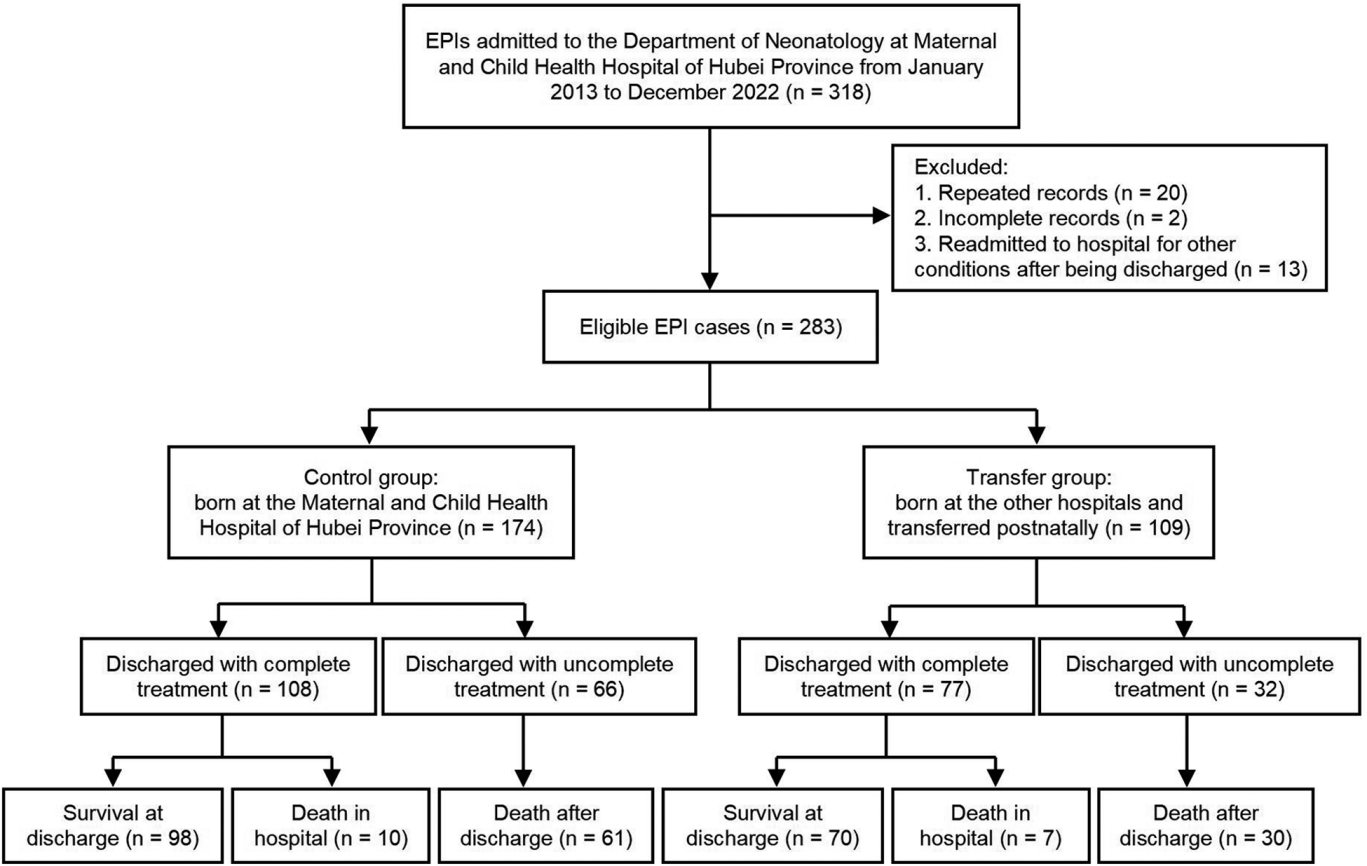
The protocol and procedure for this study.

A total of 283 EPIs were included in the study, including 174 and 109 cases in the control and transfer groups, respectively. The protocol and procedure of this study are shown in Figure 1. The EPIs in the transfer group came from 56 hospitals, of which one infant was transferred by helicopter at fourteen days of age, and the rest of the infants were transferred to the hospital by ambulance, with a median age of 1 (1, 5) days of life. Moreover, twelve cases (11.01%) were transferred after 30 days of life due to severe complications.

There was no statistical difference in neonatal gestational age, sex, small for gestational age, *in vitro* fertilization, or admission temperature between the two groups (all *P* > 0.05). The EPIs in the transfer group had a higher birth weight (1,032.66 ± 190.37 vs. 978.22 ± 165.88, *P* = 0.012), and a lower multiple pregnancy rate than those in the control group (*P* < 0.05). In the transfer group, the average admission temperature of the EPIs transferred within 24 h of birth (*n* = 67) was 36.02 ± 1.13°C, and the proportion of moderate to severe admission hypothermia was approximately 44.78% (30/67), both of which were not statistically different compared with the control group (35.79 ± 0.95, *P* = 0.057; 47.70%, *P* = 0.684) (Table 1).

**Table 1:** Characteristics of the EPIs in the study.

Parameters	Control group (*n* = 174)	Transfer group (*n* = 109)	*t*/chi-square value	*P-*value
Gestational age (weeks), mean ± SD	26.44 ± 0.76	26.42 ± 0.84	0.212	0.832
Birth weight (g), mean ± SD	978.22 ± 165.88	1,032.66 ± 190.37	−2.527	0.012*
<10th percentile (SGA) (*n*, %)	5 (2.87%)	1 (0.92%)	0.473^a^	0.492
>90th percentile (LGA) (*n*, %)	5 (2.87%)	10 (9.17%)	5.301	0.021*
Sex (male) (*n*, %)	113 (64.94%)	67 (61.47%)	0.350	0.554
*In vitro* fertilization (*n*, %)	57 (32.76%)	27 (24.77%)	2.049	0.152
Multiple pregnancy (*n*, %)	78 (44.83%)	34 (31.19%)	5.210	0.022*
Admission temperature (°C), mean ± SD	35.79 ± 0.95	36.02 ± 1.13	−1.914^b^	0.057
Moderate to severe hypothermia on admission (*n*, %)	83 (47.70%)	30 (44.78%)	0.166^b^	0.684

SGA, small for gestational age; LGA, large for gestational age.

^a^ Continuous-correction chi-squared test.

^b^ In the transfer group, the admission temperature and the incidence of moderate to severe admission hypothermia were measured in the EPIs transferred within 24 h after birth (*n* = 67).

*Statistically significant at *P* < 0.05.

### 3.2 Maternal perinatal characteristics

There was no statistical difference in the maternal age and the proportions of primiparas, gestational hypertension, abnormal umbilical cord, abnormal placenta or fetal distress between the two groups (all *P* > 0.05). However, the proportions of maternal antenatal steroids, antenatal magnesium sulfate, cesarean delivery, preterm rupture of membranes ≥18 h, gestational diabetes, and amniotic fluid abnormalities were lower in the transfer group than in the control group (all *P* < 0.05). Additionally, four EPIs in the transfer group were born at home or on the way to the hospital, whereas all EPIs in the control group were born in the hospital (Table 2).

**Table 2:** EPI maternal perinatal information.

Parameters	Control group (*n* = 174)	Transfer group (*n* = 109)	*t*/chi-square value	*P*-value
Maternal age (years), mean ± SD	31.37 ± 4.76	30.50 ± 5.87	1.351	0.178
Antenatal steroids (*n*, %)	133 (76.44%)	40 (36.70%)	44.542	<0.001*
Cesarean delivery (*n*, %)	41 (23.56%)	10 (9.17%)	9.392	0.002*
Antenatal magnesium sulfate (*n*, %)	94 (54.0%)	45 (41.3%)	4.351	0.037*
Delivery at home or on the way to the hospital (*n*, %)	0 (0.00%)	4 (3.67%)	4.111^a^	0.043*
Primipara (*n*, %)	71 (40.80%)	41 (37.61%)	0.285	0.593
PROM ≥ 18 h (*n*, %)	48 (27.59%)	14 (12.84%)	8.513	0.004*
Gestational diabetes mellitus (*n*, %)	31 (17.82%)	10 (9.17%)	4.040	0.044*
Gestational hypertension (*n*, %)	10 (5.75%)	6 (5.50%)	0.007	0.931
Abnormal amniotic fluid (*n*, %)	44 (25.29%)	16 (14.68%)	4.515	0.034*
Abnormal umbilical cord (*n*, %)	19 (10.92%)	12 (11.01%)	0.001	0.981
Abnormal placenta (*n*, %)	35 (20.11%)	17 (15.60%)	0.912	0.339
Fetal distress (*n*, %)	18 (10.34%)	6 (5.50%)	2.023	0.155

PROM, premature rupture of membrane.

^a^Continuous-correction chi-square test.

*Statistically significant at *P* < 0.05.

### 3.3 The morbidity and mortality of the EPIs

Notably, the EPIs in the transfer group had lower 1-min and 5-min Apgar scores than those in the control group (all *P* < 0.001). Additionally, EPIs in the transfer group had significantly higher rates of severe birth asphyxia (34.86% vs. 13.22%, *P* < 0.001), PIVH (42.20% vs. 29.89%, *P* = 0.034, including severe and nonsevere cases) and EUGR (17.43% vs. 6.32%, *P* = 0.003) than those in the control group. Six enrolled infants with SGA at birth all subsequently developed EUGR at discharge. Moreover, the incidences of severe PIVH (grade Ⅲ–Ⅳ), severe ROP (stage Ⅱ–Ⅲ), stage Ⅱ–Ⅲ NEC, and sBPD were slightly higher than in the control group but without statistical significance (all *P* > 0.05). No statistical difference was found in the proportions of complete treatment (62.07% vs. 70.64%), survival at discharge (56.32% vs. 64.22%), and in-hospital mortality (5.75% vs. 6.42%) between the control and transfer groups (*P* > 0.05) (Table 3).

**Table 3:** Morbidity and mortality of the EPIs.

Parameters	Control group (*n* = 174)	Transfer group (*n* = 109)	*t*/chi-square value	*P*-value
1-min Apgar score, mean ± SD	6.26 ± 2.12	4.97 ± 2.46	4.347	<0.001*
5-min Apgar score, mean ± SD	8.25 ± 1.30	6.49 ± 2.20	7.122	<0.001*
Severe birth asphyxia (*n*, %)	23 (13.22%)	38 (34.86%)	18.568	<0.001*
Sepsis (*n*, %)	39 (22.41%)	25 (22.94%)	0.010	0.919
EUGR (*n*, %)	11 (6.32%)	19 (17.43%)	8.728	0.003*
Metabolic bone disease (*n*, %)	2 (1.15%)	5 (4.59%)	2.013^a^	0.156
Severe congenital malformations or genetic diseases (*n*, %)	13 (7.47%)	6 (5.50%)	0.414	0.520
PIVH (*n*, %)	52 (29.89%)	46 (42.20%)	4.491	0.034*
PIVH rade Ⅲ–Ⅳ (*n*, %)	20 (11.49%)	13 (11.93%)	0.012	0.912
BPD (*n*, %)	97 (88.18%)	66 (86.84%)	0.074^b^	0.785
Severe BPD (*n*, %)	22 (20.00%)	16 (21.05%)	0.031^b^	0.861
ROP (*n*, %)	61 (35.06%)	46 (42.20%)	1.455	0.228
ROP grade Ⅱ–Ⅲ (*n*, %)	35 (20.11%)	27 (24.77%)	0.849	0.357
NEC (*n*, %)	15 (8.62%)	11 (10.09%)	0.174	0.677
NEC grade Ⅱ–Ⅲ (*n*, %)	6 (3.45%)	6 (5.50%)	0.698	0.403
Discharged with complete treatment (*n*, %)	108 (62.07%)	77 (70.64%)	2.176	0.140
Survival at discharge (*n*, %)	98 (56.32%)	70 (64.22%)	1.733	0.188
Death (*n*, %)	71 (40.80%)	37 (33.94%)	1.336	0.248
Death after discharge (*n*, %)	61 (35.06%)	30 (27.52%)	1.744	0.187
Death in hospital (*n*, %)	10 (5.75%)	7 (6.42%)	0.054	0.816

EUGR, extrauterine growth retardation; PIVH, periventricular-intraventricular hemorrhage; BPD, bronchopulmonary dysplasia; ROP, retinopathy of prematurity; NEC, necrotizing enterocolitis.

^a^ Continuous-correction chi-square test.

^b^ The incidence of BPD was measured in EPIs who survived at 28 days of age (110 cases in the control group, 76 cases in the transfer group).

*Statistically significant at *P* < 0.05.

### 3.4 The treatment of EPIs who survived at discharge with complete treatment

There were 98 and 70 cases in the control group and the transfer group, respectively, who survived at discharge with complete treatment,. A lower rate of utilization of surfactant replacement therapy (SRT) was found in the transfer group (70.00% vs. 93.88%, *P* < 0.001). Meanwhile, no significant difference was found between the two groups in the duration of invasive or non-invasive ventilation, parenteral nutrition, antibiotics or the rate of surgical treatments (all *P* > 0.05). See Table 4. In addition, seven infants were discharged with oxygen, and three infants were discharged with fistula bags after enterectomy. All of them were successfully deoxygenated or the fistula closed at follow-up.

**Table 4:** The treatment of EPIs who survived at discharge with complete treatment.

Parameters	Control group (*n* = 98)	Transfer group (*n *= 70)	*t*/chi-square value	*P-*value
Length of stay (days), mean ± SD	83.38 ± 22.19	77.96 ± 26.89	1.428	0.155
Days of invasive ventilation, mean ± SD	16.15 ± 17.25	14.93 ± 17.05	0.456	0.649
Days of non-invasive ventilation, mean ± SD	44.64 ± 20.43	40.17 ± 21.91	1.357	0.177
Days of parenteral nutrition, mean ± SD	41.86 ± 18.52	40.89 ± 19.08	0.331	0.741
Days of antibiotic use, mean ± SD	26.79 ± 15.70	28.97 ± 15.59	−0.892	0.374
Blood product transfusion times, mean ± SD	4.74 ± 3.81	4.86 ± 5.35	−0.159	0.874
Utilization of SRT (*n*, %)	92 (93.88%)	49 (70.00%)	17.260	<0.001*
Surgical treatment (*n*, %)	21 (21.43%)	18 (25.71%)	0.421	0.517

SRT, surfactant replacement therapy. All time indicators in the table include time from other hospitals.

*Statistically significant at *P* < 0.05.

## 4 Discussion

In this manuscript, we conducted a real-world observational study and found that EPIs born outside a provincial tertiary perinatal center and transferred postnatally had a higher rate of severe birth asphyxia, PIVH and EUGR than inborn ones. Our study also indicated that the lower proportions of antenatal steroids, antenatal magnesium sulfate, and utilization of SRT in the transfer group may be partly related to the high morbidity, but this causal relationship needs further prospective studies to verify.

The clinical care of EPIs is comprehensive and multidisciplinary, while their outcomes may reflect the regional medical level to some extent. The overall survival rate of EPIs in this study was about 59.36% (168/283), which was similar to a Chinese multicenter study among tertiary NICUs (62.3%) (3), but still lower than some developed countries during the same period. For instance, the survival rate of EPIs in Japan from 2003 to 2016 was approximately 88.4% (22), in the United States from 2013 to 2018 was approximately 78.3% (23), and in France from 2011 to 2013 was approximately 72.9% (2). Although clinical guidelines have recommended that pregnant women of low gestational age and at risk of preterm delivery should be in-utero transfer to tertiary perinatal centers, many EPIs are still delivered in lower-level hospitals and receive long-term treatment (4). In our transfer group, 38.53% of EPIs were transferred after 24 h of birth, and 11.00% of them were transferred after 30 days of birth. This suggesting that lower-level hospitals have little enthusiasm for transferring EPIs.

Previous studies reported that EPIs born in non-tertiary hospitals had higher mortality rates and rates of severe brain injury, NEC, focal intestinal perforation and cognitive impairment than the EPIs transferred *in utero* to tertiary hospitals (8–10). Severe PIVH is prone to complications of hydrocephalus, which are difficult to treat and most commonly result in cerebral palsy and neurodevelopmental delay (24). Thus, severe PIVH is the most common reason leading to the decision to withdraw EPI support (25, 26). In this study, the incidence of severe PIVH (11.66%, 33/283) is consistent with the previous studies (5%–52% globally) (3, 23, 25, 27), but no statistical difference was found between the two groups which might be due to the small sample size. Additionally, the higher incidence of PIVH (42.20% vs. 29.89%) in the transfer group may be caused by multiple factors such as inadequate resuscitation (lower 1-min and 5-min Apgar scores), insufficient neuroprotective measures (lower rate of antenatal magnesium sulfate), and stimulation during transfer. Notably, we found that the incidence of severe birth asphyxia was significantly higher in the transfer groups than in the control group (34.86% vs. 13.22%), which may cause long-term neurodevelopmental disorders and needs further study.

BPD is one of the most common severe complications of EPIs leading to prolonged hospitalization (28). The incidence of BPD is about 88.18% and 86.84% in the control and transfer groups, respectively, which is consistent with the previous studies (10%–89% globally, 10%–73% in Europe, 18%–89% in North America, 18%–82% in Asia, and 30%–62% in Oceania) (29). Neonatal respiratory distress syndrome (NRDS) is a universal disease in ELBWIs in the early stages of life, requiring mostly SRT and mechanical ventilation (30). Furthermore, severe NRDS, lack of antenatal steroids and SRT are the risk factors for sBPD (28). However, with the lower proportions of antenatal steroids (36.70% vs. 76.44%) and utilization of SRT (70.00% vs. 93.88%) in the transfer group, no significantly higher rate of sBPD was found. This may be due to confounding factors such as differences in maternal and neonatal characteristics between the two groups. Moreover, considering that cesarean delivery is associated with an increased need for SRT (31), the lower utilization of SRT in the transfer group might be partly due to the lower proportion of cesarean deliveries. Additionally, the low rate of antenatal steroids may suggest insufficient cooperation between obstetricians and neonatal care in non-tertiary hospitals. A few measures have been implemented in our NICU to improve perinatal collaborative practice, including weekly communication meetings between obstetricians and neonatal care, joint discussions of cases with high risk or poor prognosis, and monthly quality improvement meetings. These measures would serve as a reference for non-tertiary hospitals.

EUGR is influenced by a number of factors and is important for the long-term outcomes of EPIs (32). It has been reported that periods of inadequate nutrition, feeding intolerance, and morbidities associated with preterm birth may increase the risk of EUGR (32). In this study, we could not compare the details of nutritional support between the two groups because of the incomplete feeding information of EPIs in the transfer group. Therefore, considering the limitations of this study, more well-designed and large-sample studies are needed to verify our conclusions.

The limitations of the study are as follows: first, as a retrospective study, it failed to carry out homogeneous quality control of treatment measures, which may lead to bias. Also, non-tertiary hospitals were selected to transfer the critical cases that they were not capable of managing, which led to selection bias. Second, the differences in the neonatal and maternal characteristics between the two groups were allowed without propensity score matching in the real-world study, which may lead to confounding factors affecting the outcome indicators. Third, considering the regional differences in the management of EPIs, the results of this study may lack universality. Fourth, the unavailability of umbilical cord blood gas in several non-tertiary hospitals, although the causes of low Apgar scores other than birth asphyxia were excluded, may still cause a potential expansion of the diagnosis of birth asphyxia in these hospitals. Finally, since this study had a long time span, the majority of the EPIs in the study were born when delayed cord clamping was not yet available in clinical practice, so we did not provide the relevant data.

## 5 Conclusion

EPIs born outside a provincial tertiary perinatal center and transferred postnatally do not have significantly higher mortality and rates of severe complications (severe PIVH, severe ROP, and sBPD), but there may be an increased risk of severe asphyxia, PIVH and EUGR. This may be caused by differences in maternal and neonatal characteristics (such as maternal disease, neonatal birth weight, and severe birth asphyxia) and management (such as antenatal medication and neonatal utilization of SRT). Further follow-up is needed to compare neurodevelopmental outcomes, and it is recommended that EPIs be transferred *in utero* as early as possible to reduce the risk of poor physical and neurological development. Moreover, the perinatal management of EPIs, including cooperation between obstetricians and neonatal care, resuscitation in the delivery room, neuroprotection and nutritional support in the NICU, needs to be further improved in non-tertiary hospitals. Continuous quality improvement should be emphasized in all NICUs, with additional opportunities prior to high-risk mother-infant transfers.

## Data Availability

The raw data supporting the conclusions of this article will be made available by the authors, without undue reservation.
